# Professional stress-related disorders in first-line responders- how far are we from real prevention strategies?

**DOI:** 10.1192/j.eurpsy.2021.1219

**Published:** 2021-08-13

**Authors:** O. Vasiliu

**Affiliations:** Psychiatry, University Emergency Central Military Hospital Dr. Carol Davila, Bucharest, Romania

**Keywords:** professional disorders, stress-related disorders, prevention strategies, COVID-19

## Abstract

**Introduction:**

In the context of COVID-19 pandemic, first-line responders (FLR) are exposed to multiple stress factors, ranging from lack of adequate protective equipment to worries about family health due to work-related exposure to the new coronavirus. Therefore, FLR became themselves a vulnerable population that need prevention strategies for professional stress-related disorders (PSRD).

**Objectives:**

To explore the literature in order to find evidence-based prevention strategies for PSRD in FLR, strategies resulted from other epidemiological crisis situations (MERS-CoV, H1N1, SARS-CoV) that may be applied in the current pandemic.

**Methods:**

A literature review was performed through the main electronic databases (PubMed, CINAHL, SCOPUS, EMBASE) using the search paradigm “professional stress-related disorders” AND “first line responders” AND “prevention”. All papers published between January 2000 and June 2020 were included.

**Results:**

Reported prevalence of post-traumatic stress disorder in FLR involved in epidemiological crises was between 10% and 33%. Evidence-based recommendations for PSRD prevention are lacking, and only general advices have been detected. These suggestions were clustered on institutional level (e.g., involving of medical personnel in administrative decisions, encouraging personal initiatives, longer pauses between shifts) and individual level (e.g., training of coping abilities, relaxation techniques, and peer-focused group support). Several guidelines for prevention of mental disorders in workplace exist, but they are not focused on FLR.

**Conclusions:**

The need to elaborate guidelines for prevention of PSRD in FLR can not be overemphasized, especially in the pandemic period, in order to avoid the onset of stress-related complications, and to preserve a good quality of the medical activity.

